# Think Outside the Heart: An Unusual Cause of Large Hemopericardium

**DOI:** 10.14797/mdcvj.1276

**Published:** 2023-09-07

**Authors:** Nabel Rajab Basha, Priscilla Wessly, Mohammed A. Chamsi-Pasha

**Affiliations:** 1 College of Medicine, Alfaisal University, Riyadh, Riyadh Province, Kingdom of Saudi Arabia; 2 Houston Methodist DeBakey Heart & Vascular Center, Houston Methodist Hospital, Houston, Texas, US

**Keywords:** invasive thymoma, pericardial effusion, hemorrhagic pericardial effusion, mediastinal mass

## Abstract

Pericardial effusions secondary to tumors are commonly metastatic, originating primarily from the lung, breast, and lymphomas. Pericardial tamponade is a rare oncological emergency warranting early identification and treatment. We describe a 66-year-old male found to have a large bloody pericardial effusion causing tamponade physiology, and multimodality imaging was consistent with intrapericardial malignancy with no identifiable primary source. He was subsequently diagnosed with type B3 thymoma after mediastinal resection.

## Introduction

Although hemopericardium can have multiple etiologies, most result from inflammation and malignancy or after cardiotomy. In cancer patients, 1 out of 10 patients can have malignant pericardial involvement, with the most common tumors being breast, lung, and lymphomas.^1,2^ Thymoma is a rare neoplasm originating from the thymic epithelium, with an incidence of 0.13 per 100,000 person-years, and is identified incidentally on imaging in asymptomatic patients.^3^ Rarely, a pericardial effusion may be the initial manifestation of thymoma, and in severe cases it can result in cardiac tamponade.^4^

## Case Presentation

**Figure 1:**
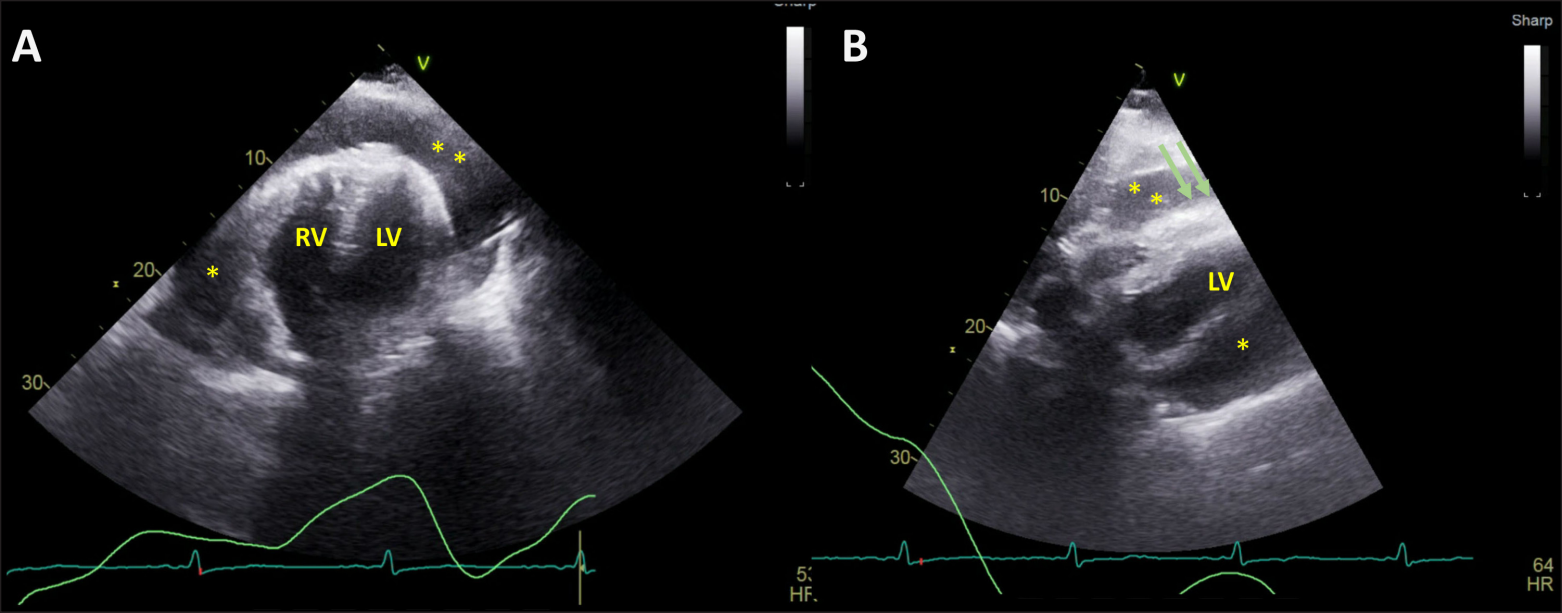
Transthoracic echocardiography, apical 4-chamber **(A)** and subcostal view **(B)** showing large circumferential pericardial effusion (asterisks) with right ventricular diastolic collapse (green arrows). LV: left ventricle; RV: right ventricle

A 66-year-old male patient with a past medical history of morbid obesity, hypertension, and diabetes presented to the clinic with progressive dyspnea on exertion, paroxysmal nocturnal dyspnea, and orthopnea. In addition, the patient recalls a 70-pound weight loss over the course of 4 months. Physical examination revealed a morbidly obese patient (BMI 43 kg/m^2^) with a normal heart rate and respiratory rate. It was difficult to assess the jugular vein due to his body habitus. His blood pressure was 138/81, and he had distant heart sounds on auscultation. A 12-lead electrocardiogram showed normal sinus rhythm with low voltage QRS. An echocardiogram to evaluate cardiac causes of dyspnea showed a large circumferential pericardial effusion with features of elevated intrapericardial pressures (right ventricular diastolic collapse and respiratory variation across the mitral inflow) (Figure 1, Video 1).

**Video 1:** Transthoracic echocardiography with Doppler showing hemodynamically significant large pericardial effusion with right-sided chamber collapse and respiratory variation across the inflow; see also at https://youtu.be/VI1MBbjn2Vw.

As a result, the patient was hospitalized and underwent next-day pericardiocentesis with the removal of 1,850 mL of bloody fluid and an additional 1,355 mL through a pericardial drain. Fluid analysis revealed a lactate dehydrogenase of 382 IU/L, protein count of 4.8 g/dL, and cell count with largely bloody fluid (949,000 red blood cells, 900 nucleated cells, 69% lymphocytes, and 29% macrophages). Cultures including bacterial, acid-fast bacilli, and fungal as well as cytology were negative for infectious or malignant cells. Laboratory studies showed normal complete blood counts (hemoglobin 14 g/dL, white cell counts 5.63 K/uL), and normal liver and renal function tests with mildly elevated C-reactive protein of 3.63 mg/dL.

**Figure 2:**
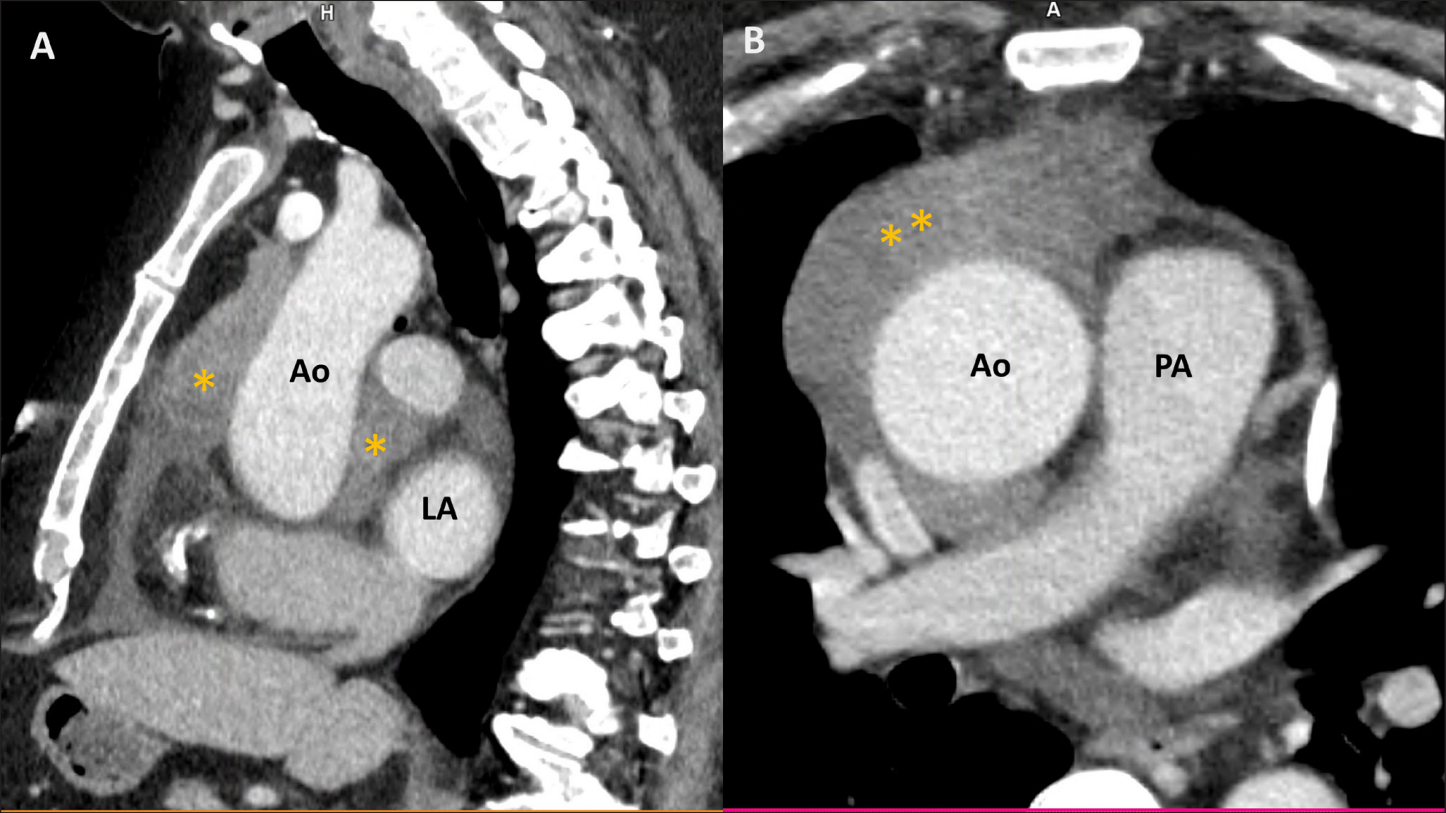
Contrast-enhanced computed tomography scan of the chest, sagittal view **(A)**, and axial view **(B)** showing a hyperintense (Housefield unit of 50) large lobulated anterior mediastinal mass (asterisks) encasing the aorta and extending posteriorly to the left atrium. Ao: aorta; LA: left atrium; PA: pulmonary artery

**Figure 3:**
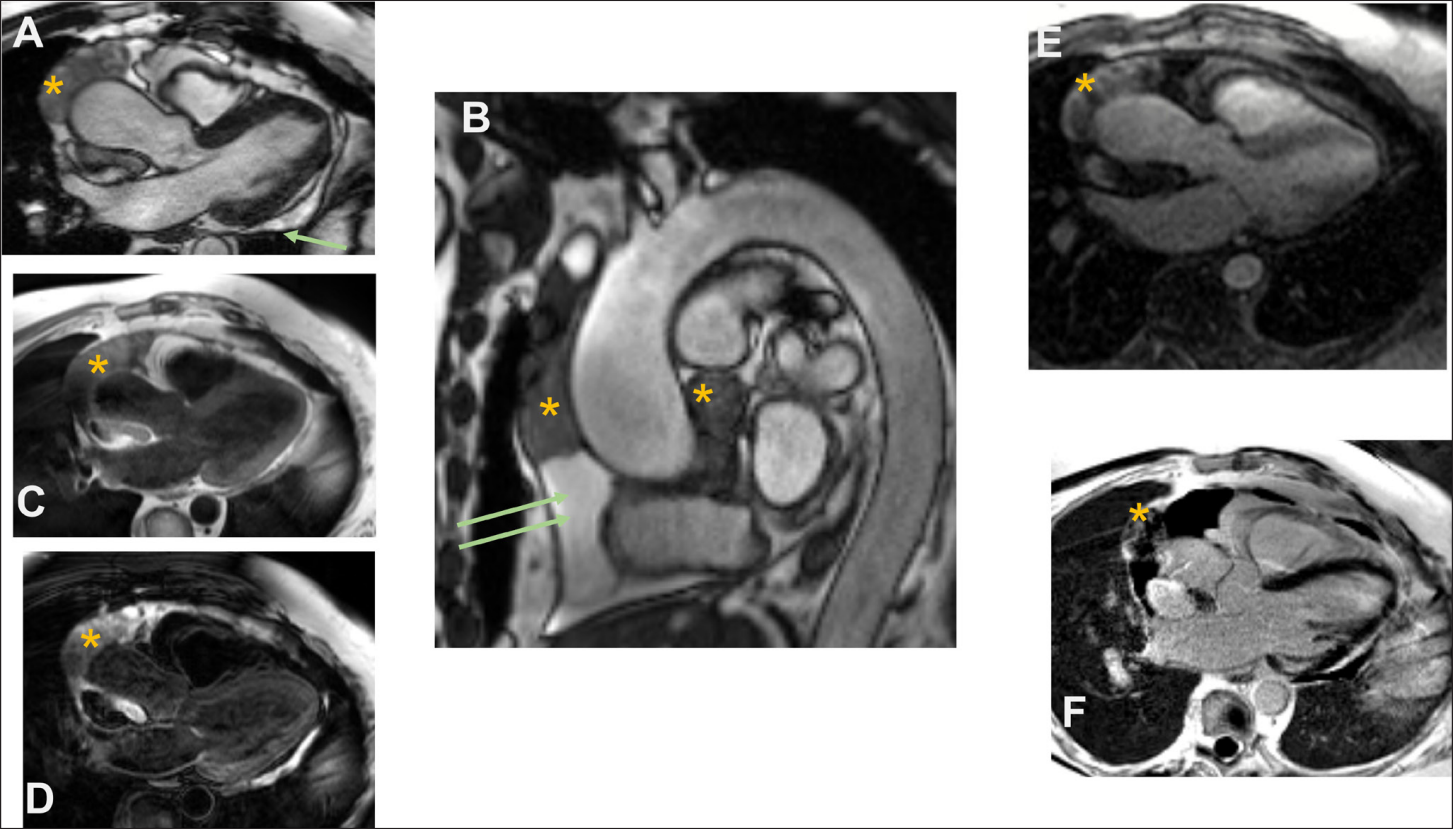
Cine cardiac magnetic resonance imaging. **(A)** 3-chamber view showing heterogenous lobulated intrapericardial mass (asterisk) with a posterior pericardial effusion (arrow). **(B)** Sagittal candy-cane view of the aorta showing pericardial effusion (arrows) and masses (asterisk). **(C-D)** T1- and T2-weighted imaging showing isointensity on T1 and hyperintensity on T2. **(E-F)** First pass perfusion and late gadolinium imaging showing evidence of contrast uptake on the first pass and heterogenous signal intensity suggesting patchy late gadolinium uptake.

Given constitutional symptoms of weight loss and bloody effusion, additional imaging was recommended to evaluate primary cancerous etiology. Computed tomography (CT) of the chest with contrast revealed a large (7.7 × 2.8 × 8.5 cm) lobulated, heterogeneous, slightly hyperdense anterior mediastinal mass (Figure 2). The patient was started on high-dose ibuprofen and colchicine for the treatment of pericarditis, with improvement of symptoms. Cardiac magnetic resonance imaging (CMR) for better tissue characterization showed a large lobulated intrapericardial mass encasing the ascending aorta, extending superiorly to the level of the arch and posteriorly to the left atrium. A moderate-sized effusion was present. The mass was isointense on T1-weighted imaging and hyperintense on T2-weighted imaging. There was evidence of contrast uptake on first-pass perfusion along with gadolinium enhancement on delayed imaging (Figure 3, Videos 2, 3). The differential was for vascular tumors such as paraganglioma, sarcomas, or hemangiomas. There was no evidence of inflammation of the pericardium to suggest pericarditis.

**Video 2:** Noncontrast, axial bright blood imaging showing large heterogenous intrapericardial mass extending superiorly to the arch and encasing the aorta; see also at https://youtu.be/pnJ-g_lxM0c.

**Video 3:** First pass perfusion with cardiac magnetic resonance showing contrast uptake; see also at https://youtu.be/3qS6IYbRYxk.

After referring the patient to the endocrinology clinic for neuroendocrine tumor workup, a comprehensive biochemical workup was performed, including chromogranin A, plasma-fractionated catecholamines, plasma-free metanephrines, and NETSPOT ^68^Ga-dotatate positron emission tomography/CT (NETSPOT PET/CT) scan, all with no radiographic or biochemical evidence of neuroendocrine tumor.

**Figure 4:**
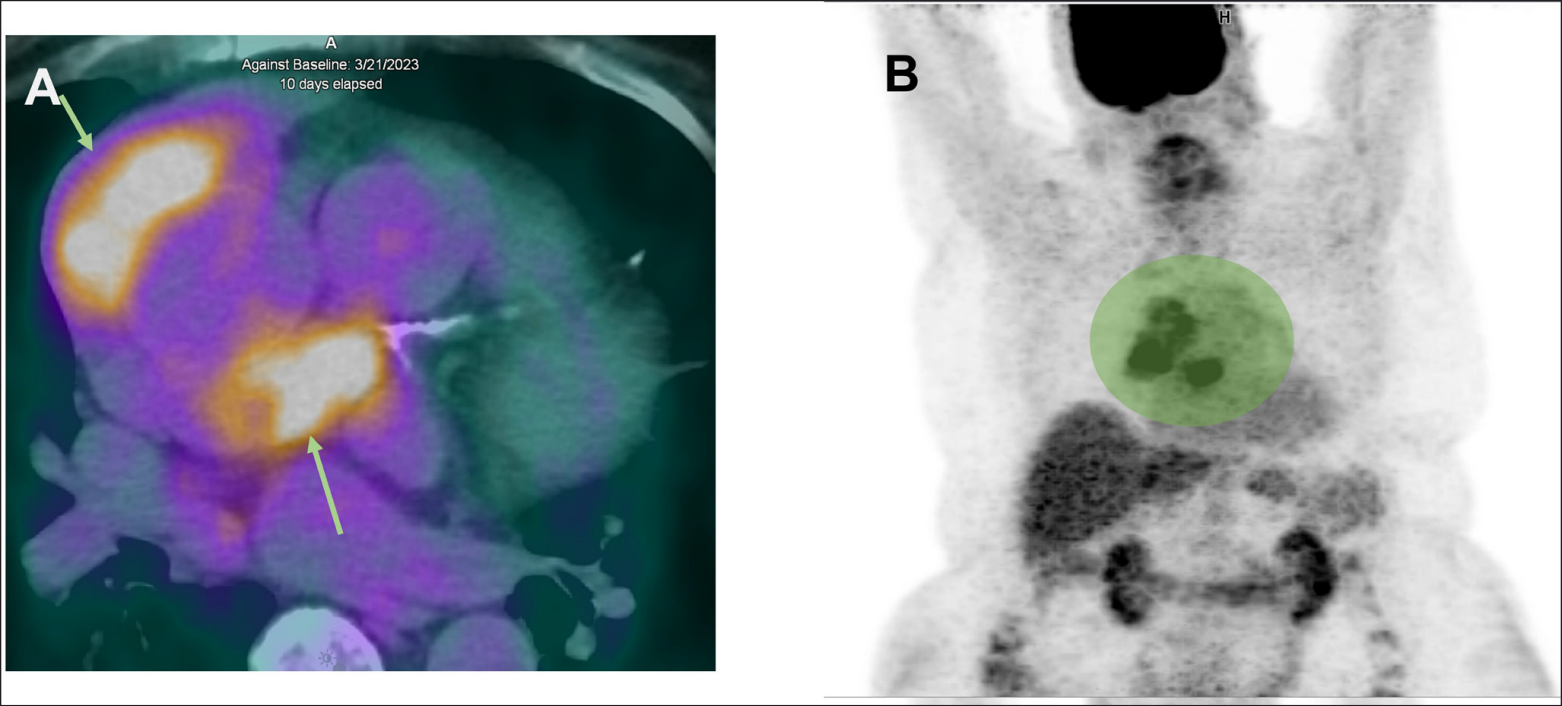
**(A)** Fluorine-18-fluorodeoxyglucose (18F-FDG) positron emission tomography/computed tomography shows positive FDG uptakes in periaortic pericardial masses (arrows). **(B)** Corresponding whole body PET/CT imaging with green area showing increased metabolic activity and FDG uptake but no evidence of mediastinal lymph node or extracardiac metastasis.

Subsequently, the patient underwent a PET/CT using fluorine-18-fluorodeoxyglucose, which revealed an anterior mediastinal/periaortic mass demonstrating high metabolic activity, evidenced by a maximum 7.8 standardized uptake value (SUV_max_) (Figure 4). As a result, the patient underwent robotic surgical resection that showed the tumor invading the pleura and right upper lobe lung parenchyma, and hence it was not completely resectable. The pathological specimen showed sheets of polygonal epithelial cells with mild to moderate cellular atypia, and immunohistochemical stains were positive for CK5/6 and P40. The final diagnosis was type B3 thymoma (a high-risk thymic neoplasm) according to the World Health Organization Classification of Thymic Epithelial Tumors.^5^ The patient is currently undergoing radiation therapy.

## Discussion

Thymomas are a rare malignant neoplasm of thymic epithelial origin located in the anterior mediastinum. They account for 255 of all mediastinal tumors with an incidence of 0.13 per 100,000 person-years. Usually seen in adults aged 45 to 50 years, the tumor has variable clinical presentation ranging from being incidentally detected during chest imaging to causing symptoms (cough, chest pain, dyspnea) due to direct invasion into surrounding organs like pleural, pericardial space or lungs, or mass effect.^6,7,8^ Pericardial effusion is an established complication of malignancy, which can occur as a direct consequence of malignancy itself or as an adverse effect of treatment.^1,9^ Pericardial tamponade complicating thymoma is rare, with only a few published case reports over the years.^6,10,11,12^ Despite a higher yield of fluid cytology in malignant cases (50-80%), prior reports of thymoma invading pericardial space have shown nondiagnostic fluid analysis.^6,10^

The multimodality imaging approach to this case was essential for clinical decision-making and prognostication. However, significant overlap occurs among different thymic tumors, which can limit diagnostic accuracy.^13^ Usually, thymomas are smooth and homogeneous on CT imaging, unlike our case that had more heterogeneity and irregular/lobulated contour.^14^ Cine-CMR has an unrestricted field of view with superior tissue characterization utilizing gadolinium contrast when compared to CT.^15^ It has been studied in evaluating benign from malignant cardiac neoplasms, with the presence of pericardial effusion, first-pass perfusion, and late gadolinium enhancement all supportive of the malignant etiology.^16^

PET/CT using fluorine-18-fluorodeoxyglucose (^18^F-FDG) identifies areas of increased metabolic activity in malignancies. A meta-analysis of 11 studies was conducted to differentiate between low-grade thymomas (type A, AB, and B1), high-grade thymomas (type B2 and B3), and thymic carcinomas (type C).^13^ The threshold proposed was SUV_max_ of 1.2 between low-risk and high-risk thymomas. Our patient’s SUV_max_ was 7.8, which was consistent with high-grade and aggressive thymoma. However, given the significant overlap of these semi-quantitative values, an absolute cutoff to discriminate different groups could not be defined.

Type B3 thymomas carry a worse prognosis compared with the lower-risk type A. The treatment may include a combination of chemotherapy, radiation, and/or surgery, with known extreme radiosensitivity of such tumors.^17^ Treatment of nonmetastatic thymoma is complete resection whenever feasible. Furthermore, ongoing research is exploring the potential of immunotherapy as an emerging treatment for thymomas. In our case of unresectable thymoma, the patient is being considered for additional chemoradiation treatments.

## Conclusion

Thymomas are rare mediastinal tumors that are largely asymptomatic and small, but on occasion it can invade intrapericardial space and result in large hemopericardium and pericardial tamponade. Multimodality imaging and a multidisciplinary team approach are needed for accurate diagnosis and better treatment outcomes.
